# Effects of Heat-Treatment Temperature on the Microstructure and Mechanical Properties of Steel by MgO Nanoparticle Additions

**DOI:** 10.3390/ma11091707

**Published:** 2018-09-13

**Authors:** Yutao Zhou, Shufeng Yang, Jingshe Li, Wei Liu, Anping Dong

**Affiliations:** 1 School of Metallurgical and Ecological Engineering, University of Science and Technology Beijing, Beijing 100083, China; zhouyutao16@163.com (Y.Z.); lijingshe@ustb.edu.cn (J.L.); youiithe@foxmail.com (W.L.); 2 School of Materials Science and Engineering, Shanghai Jiao Tong University, Shanghai 200240, China

**Keywords:** microstructure, oxide metallurgy, mechanical properties, intragranular acicular ferrite

## Abstract

The characteristics and formation mechanisms of intragranular acicular ferrite (IAF) in steel with MgO nanoparticle additions were systematically investigated for different isothermal heat-treatment temperatures, and its influence on mechanical properties was also clarified. The results indicate that the inclusions were finely dispersed and refined after adding MgO nanoparticles. In addition, with decreasing heat-treatment temperature, the microstructure changed from grain boundary ferrite (GBF) and polygonal ferrite (PF) to intragranular acicular ferrite. Moreover, the steel with MgO additions had excellent mechanical properties in the temperature range of 973 to 823 K and an average Charpy absorbed energies value of around 174 J at 873 K due to the significant refinement of the microstructure and nucleation of intragranular acicular ferrite.

## 1. Introduction

With the development of a new generation of steel materials, the cleanliness of steel has been significantly improved [1,2,3]. However, the complete removal of nonmetallic inclusions from liquid steel during the steelmaking process is almost impossible [4,5]. Therefore, Takamura et al. [6] and Mizoguchi et al. [7] proposed a new concept of oxide metallurgy, which uses small-sized inclusions and precipitates particles as nucleation sites of intragranular acicular ferrite (IAF) or pins austenite grains to improve the final properties of steel. Further research and applications of this technique have shown that nonmetallic inclusions induce the formation of IAF, which becomes the core point [8].

Up to now, these inclusions have been formed either by an internal precipitation method or by an external addition method [9]. Numerous studies have focused on the internal precipitation method, which mainly forms oxide inclusions through strong deoxidizer and alloying elements such as Mg [10,11], Ti [12,13], V [14], and Ce [15], and these inclusions become nucleation sites of IAF during the austenite (γ) → ferrite (α) transformation [16]. In addition, some researchers have suggested that inclusions such as ZrO_2_ [17], TiN [18], and MnS [19] can also act as heterogeneous nuclei for IAF. However, this method requires precise control of the refining process within a narrow operating window. Moreover, due to the complexity of the metallurgical chemical reaction and the instability of the steelmaking process, it is difficult to obtain the desired inclusion characteristics, especially the inclusion type and size. Therefore, in the past few decades, some researchers have proposed an external addition method, which mostly uses artificially added oxide powder such as Ti_2_O_3_ and ZrO_2_ to produce the desired inclusions types [20]. Kiviö et al. [21] reported that they had added to steel different additives with metallic Ti and TiO_2_. Samples brought for subsequent hot rolling and heat-treatment experiments to find out the grain-refining effect eventually formed IAF. Mu et al. [22] and Xuan et al. [23] investigated different powders such as Ti_2_O_3_ and TiN, which were used for steel samples to act as nucleation sites of IAF formation. Furthermore, recent studies have attempted to use various methods of externally adding different particles to obtain small-sized and uniformly distributed inclusions [24,25].

In the previous research of the author and his coworkers [26,27,28], a novel method for predispersing MgO nanoparticles with AlSi or AlTi alloy nanoparticles was applied. Small-sized inclusions could be obtained by adding nanoparticles to molten steel. For laboratory experiments, these nanoparticles could be added to liquid steel with a molybdenum rod in a tubular resistance furnace [26]. For industrial production, the nanoparticles could be added to liquid steel within the mold in the vicinity of the submerged nozzle outlet [27]. Meanwhile, several studies have reported the formation mechanism of IAF induced by the addition of nanoparticles. Gao et al. [28] determined that the Mn-depleted zone (MDZ) can be found around Ti_2_O_3_ and MgO–Al_2_O_3_ inclusions in steels by MgO addition, which can promote ferrite nucleation by increasing the chemical driving force. However, Mu et al. [29] reported that the MDZ was not formed around inclusions in steels by Ti_2_O_3_ addition. Similar conclusions were also drawn by Kang et al. [30]. Hence, it is of great significance to systematically investigate the formation mechanism of IAF in steel.

Although the addition of nanoparticles is very popular for the induction of IAF nucleation to improve the mechanical properties of steels, few researchers have systematically studied the nucleation of IAF and how to refine the microstructure, in particular, the effect of phase transformation temperatures on nucleation and growth of IAF during γ → α transformation. Therefore, the current study, based on previous studies, investigated the effects of isothermal treatment temperature on microstructure and mechanical properties. Then, the critical size of the inclusions for IAF nucleation was analyzed using theoretical calculations, and the mass fraction of the inclusions during solidification was predicted using FactSage software. Finally, the mechanism of IAF formation and the refinement of IAF to microstructure are discussed.

## 2. Experimental

### 2.1. Experimental Procedure

**Figure 1:**
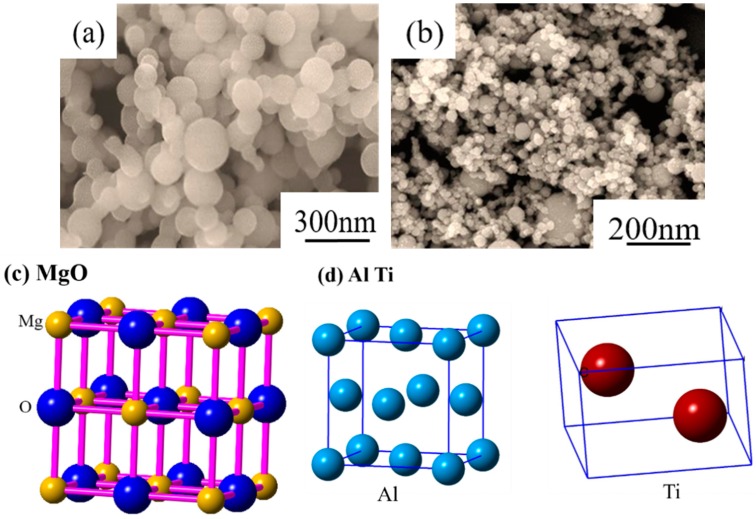
Morphology and crystal structure of MgO and AlTi nanoparticles. (**a**) Morphology of MgO nanoparticles, (**b**) morphology of AlTi nanoparticles, (**c**) crystal structure of MgO nanoparticles, and (**d**) crystal structure of AlTi nanoparticles.

The low-carbon steel used in the present study was melted in a 6-kg vacuum induction furnace (Shenyang Vacuum Technology Institute Co., Ltd., Shenyang, China), and the mixed MgO nanoparticles were poured into the liquid steel through a charging hopper located under the furnace cover. In order to ensure that the MgO nanoparticles added to the steel melt were fully dispersed, another type of nanoparticle material, AlTi alloy, was used as a predispersion medium. The predispersion process of MgO nanoparticles was performed using a planetary ball mill (IKN Mechanical Equipment Co., Ltd., Berlin, Germany) with a weight ratio of MgO and AlTi alloy of 4:1. The rotation speed was maintained at 6000 rpm during the 3-h process which was conducted under low-oxygen and low-temperature conditions. The microstructure and crystal structure of MgO and AlTi alloy nanoparticles are shown in Figure 1. The detailed operational parameters and processes of the nanoparticles have been reported by previous studies by the author and his coworkers [26]. Then, the melt was cast into ingots and was cooled to room temperature in the air. Subsequently, the ingots were heated at 1473 K for 1 h to homogenize the elements, hot forged into a rectangular shape with the size of 40 × 120 × 120 mm^3^ at a temperature ranging from 1323 to 1073 K, and then air cooled to room temperature. The chemical composition of the steel was determined using optical emission spectroscopy and is given in Table 1.

**Figure 2:**
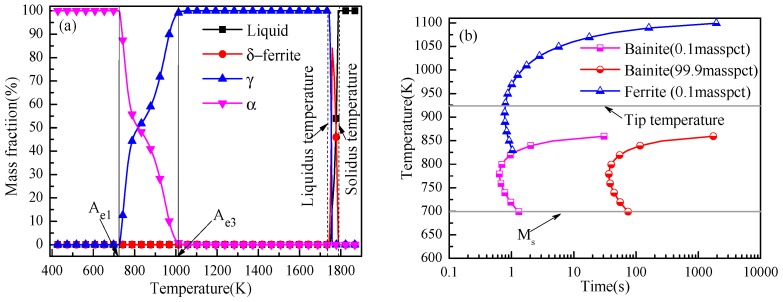
Determination of heat-treatment temperature. (**a**) The relationship between the mass fraction of each phase and the temperature in equilibrium state calculated using FactSage 7.1 software, and (**b**) TTT diagram of the experimental steel calculated using JMatPro software.

**Figure 3:**
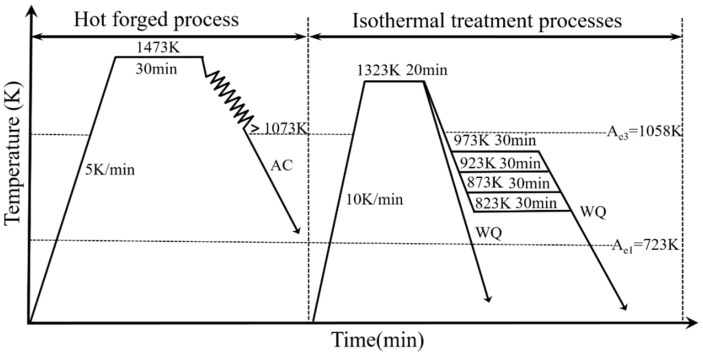
Schematic of forging process and isothermal heat treatment of specimens.

To evaluate the effect of the different phase transformation temperatures on IAF, first, thermodynamic software was used to predict the phase transformation temperatures under equilibrium conditions to provide theoretical guidance for the experiment. The phase transformation temperatures, A_e1_ and A_e3_, were calculated by FactSage7.1 software (Centre for Research in Computational Thermochemistry, Montreal, QC, Canada,) (Figure 2a) to be 723 and 1058 K, respectively. The TTT (Time, Temperature, Transformation) diagram was established by JMatPro software (Version 10.0, Sente Software Ltd., London, UK) as shown in Figure 2b, and the tip temperature was 919 K during γ → α transformation. The isothermal heat-treatment processes were as follows: Specimens with a size of 12 × 120 × 50 mm^3^ from forged steels were heated to 1323 K at a rate of 10 K/min and held at this temperature for 20 min before being cooled to the corresponding salt bath temperature at the same rate. Subsequently, the isothermal treatment temperatures based on the TTT diagram were carried out at 973, 923, 873, and 823 K for 30 min, respectively. For convenience, the specimens were orderly denoted as M1, M2, M3, and M4, respectively. Lastly, the heat-treated specimens were water-cooled to room temperature. In addition, the forged specimens were heated to 1323 K at a rate of 10 K/min and held at this temperature for 20 min, followed by water-quenching to reveal the prior austenite grain boundaries. The details schematic of the heat-treatment process is shown in Figure 3.

### 2.2. Characterization of Inclusions

Different specimens were cut into small cubes (10 × 10 × 10 mm^3^). Subsequently, they were polished by SiC papers and, finally, by 1-μm diamond pastes. Thereafter, the morphology and composition of inclusions were observed with scanning electron microscopy (SEM, Phenom proX scanning electron microscopy, Eindhoven, The Netherlands) and energy-dispersive spectroscopy (EDS) (PANalytical B.V., Eindhoven, The Netherlands). The characteristics of the inclusions were analyzed by Image-Pro-Plus software (Version 6.0, Media Cybernetics, Inc., Rockville, MD, USA).

### 2.3. Mechanical Properties and Microstructural Characteristics

Tensile specimens were machined to a length of 60 mm and diameter of 5 mm, which were conducted at room temperature using a computerized tensile testing system. Standard specimens with a size of 10 × 10 × 55 mm^3^ were machined for Charpy impact tests which were conducted at ambient temperature. The fracture surfaces were examined by SEM and the inclusions in the dimples were analyzed by EDS. All the specimens were tested by Rockwell Hardness and the values were reported as an average of five measurements to reduce the error. To investigate the microstructure characteristics by optical microscopy (OM, Leica, Wetzlar, Germany) and SEM, the polished specimens were slightly etched for 15 s with 4 vol% nital solution. In addition, the forged specimens were etched by a solution (3 g picric acid, 50 mL distilled water, and 2 mL HCl) at 333 K to reveal the prior austenite grain boundaries and then observed by OM. X-ray diffraction (XRD, Rigaku Corporation, Tokyo, Japan) patterns of all the specimens were obtained with Cu-Ka radiation.

## 3. Experimental Results

### 3.1. Characteristics of Inclusions

**Figure 4:**
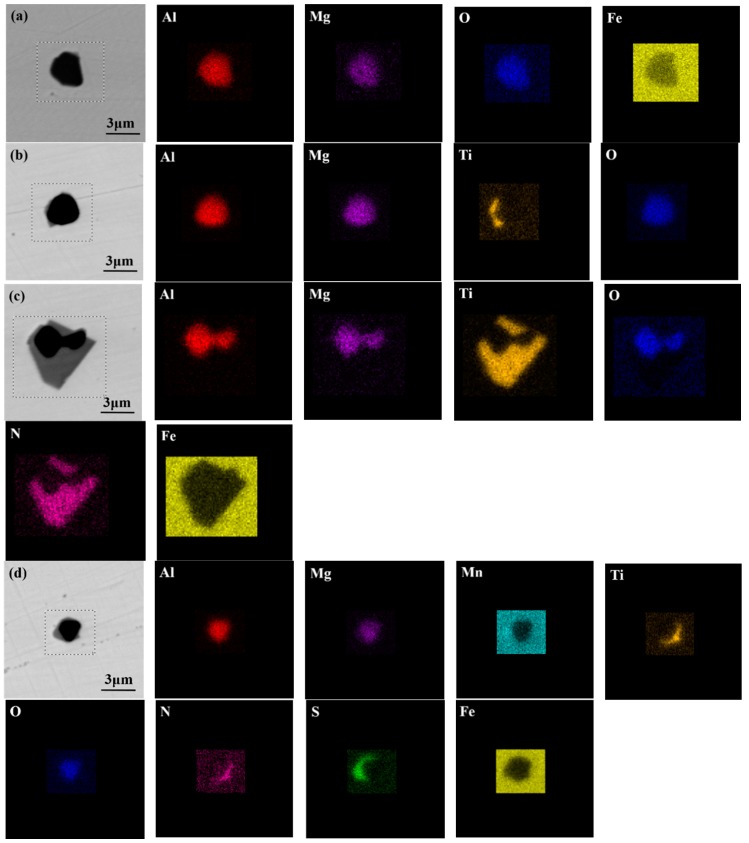
Morphology and composition of typical inclusions. (**a**) MgO–Al_2_O_3_; (**b**) MgO–Al_2_O_3_-TiO_2_; (**c**) MgO–Al_2_O_3_–TiO_2_–TiN; (**d**) MgO–Al_2_O_3_–TiO_2_–TiN–MnS.

Before studying the effect of heat-treatment temperature on the microstructure, it is necessary to clarify the inclusion characteristics in experimental steels. Figure 4 shows the elemental distribution of the typical inclusions in steel with the MgO addition. Figure 4a,b shows that the inclusions mainly consisted of MgO–Al_2_O_3_ and MgO–Al_2_O_3_–TiO_2_. The single inclusion was irregular in shape and most of the inclusions were less than 3 μm in size. Moreover, in Figure 4b, it can be seen that TiO_2_ was formed on the surface of MgO–Al_2_O_3_ inclusions and the inclusions had a spherical tendency. Figure 4c shows the complex MgO–Al_2_O_3_–TiO_2_–TiN inclusion. Based on the elemental mapping, the central area contains the elements Al, O, and Mg, and the outermost layer consists of mostly Ti and N. Figure 4d shows the complex MgO–Al_2_O_3_–TiO_2_–TiN–MnS inclusion, and MnS and TiN were found to precipitate randomly at the corners of the core of MgO–Al_2_O_3_–TiO_2_. A detailed discussion of the microstructures is presented later.

**Figure 5:**
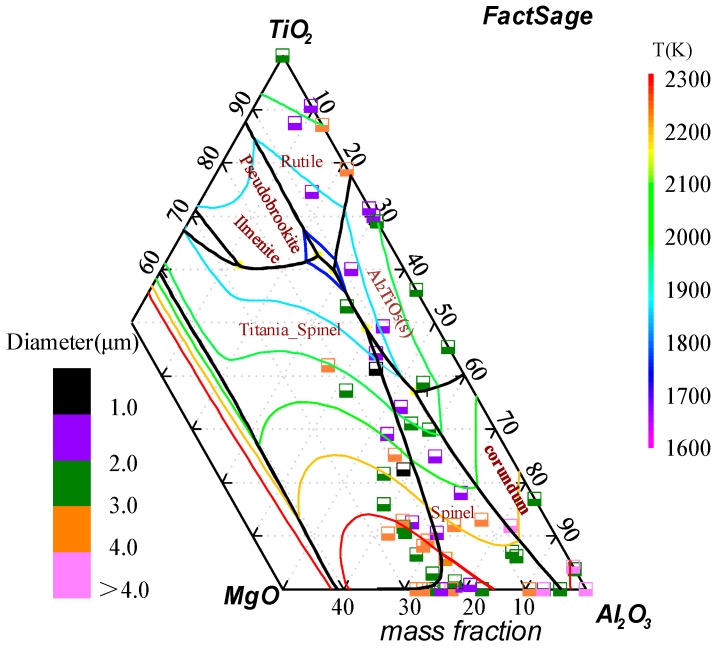
Size distributions of oxide inclusions in experimental steels.

The composition and distributions of oxide inclusions in experimental steels are shown in Figure 5. The solid lines and isotherm of the TiO_2_–MgO–Al_2_O_3_ ternary system were calculated using FactSage7.1 software, and the composition is the mass fraction and the size chart of the inclusions is represented by the color map. The percentage of inclusions smaller than 3 μm was greater than 80%, with most of the inclusions having a size between 1 and 2 μm, and the large-sized inclusions were not observed in all specimens. This indicates that the inclusions were finely dispersed and refined after adding MgO nanoparticles, which is consistent with the findings by Gao et al. [28]. Further, Lee et al. [31] found that larger inclusions have a greater potential for the nucleation of ferrite, and when the inclusion size is 1.1 μm in steel, the probability of nucleation is 1. Therefore, the size distribution of inclusions in experimental steel was optimal.

### 3.2. Microstructural Characteristics

**Figure 6:**
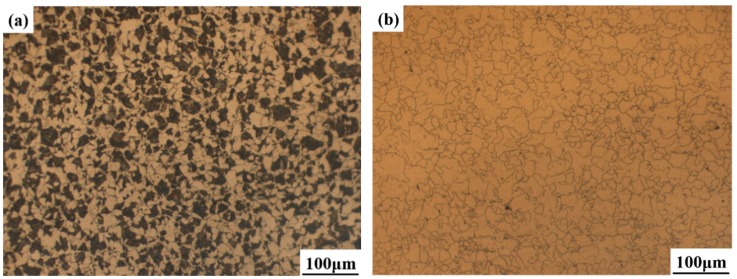
Optical micrograph of experimental steels. (**a**) Initial microstructure of steels after forging, and (**b**) prior austenite grain micrograph of experimental steels quenched at 1323 K (1050 °C).

Figure 6 displays the microstructure of the experimental steel and shows that the microstructure of the experimental steel after hot forging mainly consisted of pearlite and ferrite. The white area was ferrite and the black area was pearlite. Figure 6b presents the prior austenite grains of experimental steels quenched at 1323 K. It is clear that the prior austenite grain size was slightly fine and uniform. The mean prior austenite grain size of the specimen was measured to be 45 μm using the intercept method, which is similar to the one reported Wan et al. [32].

**Figure 7:**
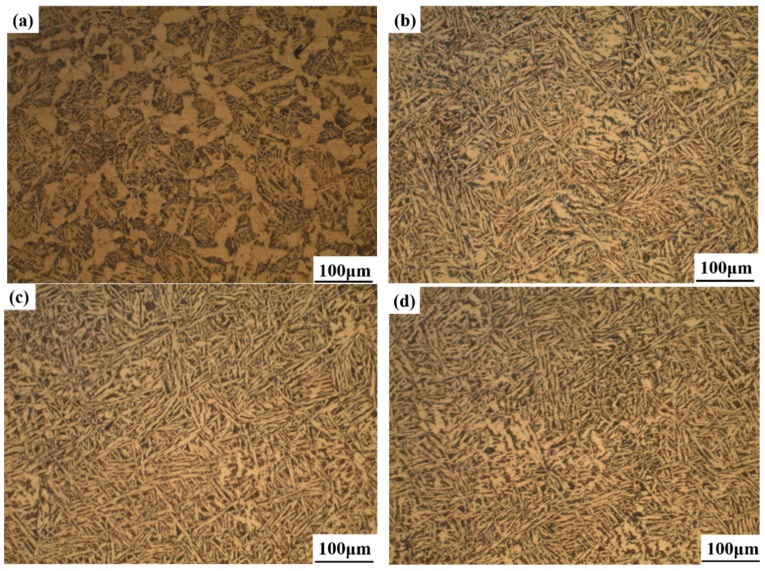
Typical microstructure of the specimens. (**a**) M1, (**b**) M2, (**c**) M3, and (**d**) M4.

Figure 7 shows the typical microstructure of the M1, M2, M3, and M4 specimens. It is seen from Figure 7a that the microstructure in M1 was composed of coarse grain boundary ferrite (GBF), polygonal ferrite (PF), and little Widmanstätten ferrite (WF). When the heat-treatment temperature was 923 K, the amounts of GBF and PF were significantly decreased, and the amount of IAF was increased (Figure 7b). Figure 7a,b shows that the PF in the latter was much finer than in the former. The microstructures of M3 and M4 are shown in Figure 7c,d, respectively. It was found that the dominant microstructure for M3 and M4 was IAF, and a small amount of bainite was also observed in M4. Although IAF existed in M4, they were much coarser than those in M3. Therefore, it can be inferred that the nucleation and growth of IAF were closely related to the isothermal heat treatment.

**Figure 8:**
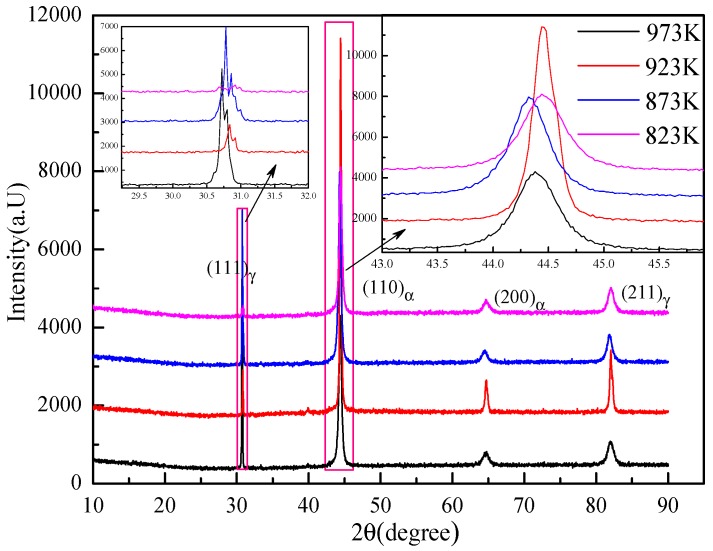
X-ray diffraction curves of the specimens.

X-ray diffraction patterns of all the specimens are shown in Figure 8. Peaks corresponding to ferrite and austenite were indexed based on respective (h k l) planes of phases. It can be clearly observed that the XRD diffraction patterns of the four specimens were basically similar. Austenite diffraction peaks were clearly present in the XRD diffractogram, but the number was small. The peak of ferrite was obvious, exhibiting two strong lines and broad half peaks at the diffraction angles of 44.4° and 64.8°. Therefore, it can be seen that ferrite should be the main phase.

**Figure 9:**
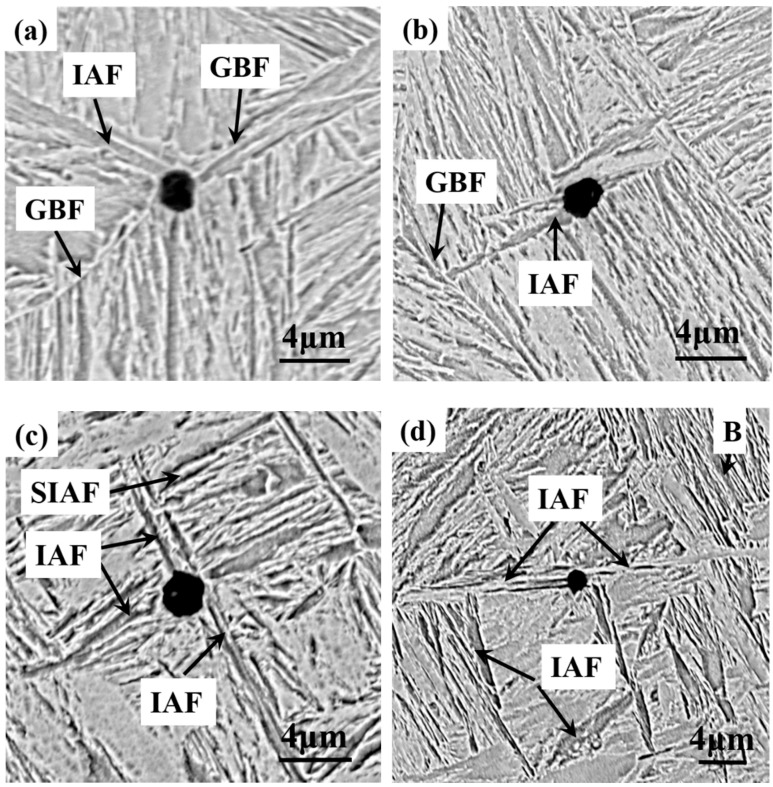
Morphologies of intragranular acicular ferrite (IAF) induced by inclusions for different specimens. (**a**) M1, (**b**) M2, (**c**) M3, and (**d**) M4.

To further investigate the IAF nucleated on inclusions at different heat-treatment temperature, the microstructure of four specimens were observed by SEM, as shown in Figure 9. Figure 9a,b shows that several PF nucleated on the surface of the small-sized inclusions and a large amount of GBF and some IAF were observed, but IAF plates were still very short, indicating that IAF transformation did not occur in the vicinity of complex inclusions after quenching at 973 and 923 K. In Figure 9c, it can be observed that IAF and PF nucleated on complex inclusions (approximately 2 μm in size). In contrast, IAF in M3 seemed to be longer, indicating that IAF had a growth in the length direction. Moreover, some secondary intragranular acicular ferrites (SIAF) were found on the branches of IAF, as indicated with arrows. The formation of SIAF might help promote the final properties of the steel by increasing the multiple interlocking degree of IAF [33]. The microstructure in M4 was composed of coarse IAF, PF, and some bainites (B), as shown in Figure 9d.

**Figure 10:**
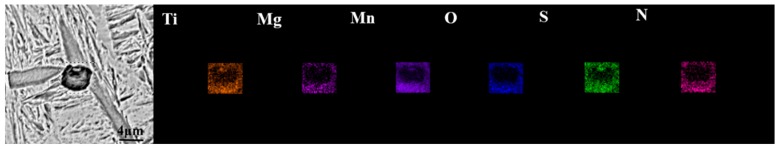
SEM mapping images of inclusion nucleating IAF in M3.

Typical elemental mapping images of inclusions inducing IAF nucleation in M3 are shown in Figure 10. It can be found that IAF preferentially nucleates at MgO–Al_2_O_3_–TiO_2_–TiN–MnS complex inclusions located within the austenite grain, and many IAF plates can be clearly observed around the inclusions, which is in good agreement with other studies [34]. The results indicate that the abovementioned inclusions are effective at providing nucleation sites for IAF at 873 K.

### 3.3. Mechanical Properties

**Figure 11:**
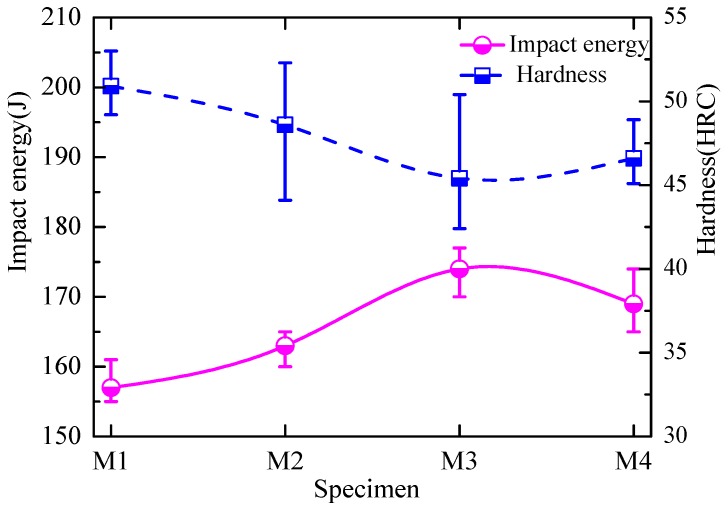
Variation of macrohardness and the Charpy absorbed energy at room temperature.

To compare the mechanical properties of the experimental steels after different heat-treatment temperatures, tensile tests were performed on the specimens and the results are shown in Table 2. It can be seen that the heat-treatment temperature had a slight effect on tensile strength and yield strength, and the yield strength and the tensile strength varied from 529 to 540 MPa and 763 to 803 MPa, respectively. Figure 11 shows the variation in the specimens’ macrohardness and the Charpy absorbed energy at room temperature. It is obvious that the macrohardness first decreased and then increased slightly as the heat-treatment temperatures decreased from 973 to 823 K. The average Charpy absorbed energy values at 298 K for specimens of M1, M2, M3, and M4 were 157, 163, 174, and 169 J, respectively, which showed excellent impact properties. The absorbed energy tended to increase in the order of specimen M1, M2, and M3 because M3 contains a large amount of IAF (Figure 7). This indicates that the formation of IAF induced by inclusions was conducive to improve toughness [35,36].

**Figure 12:**
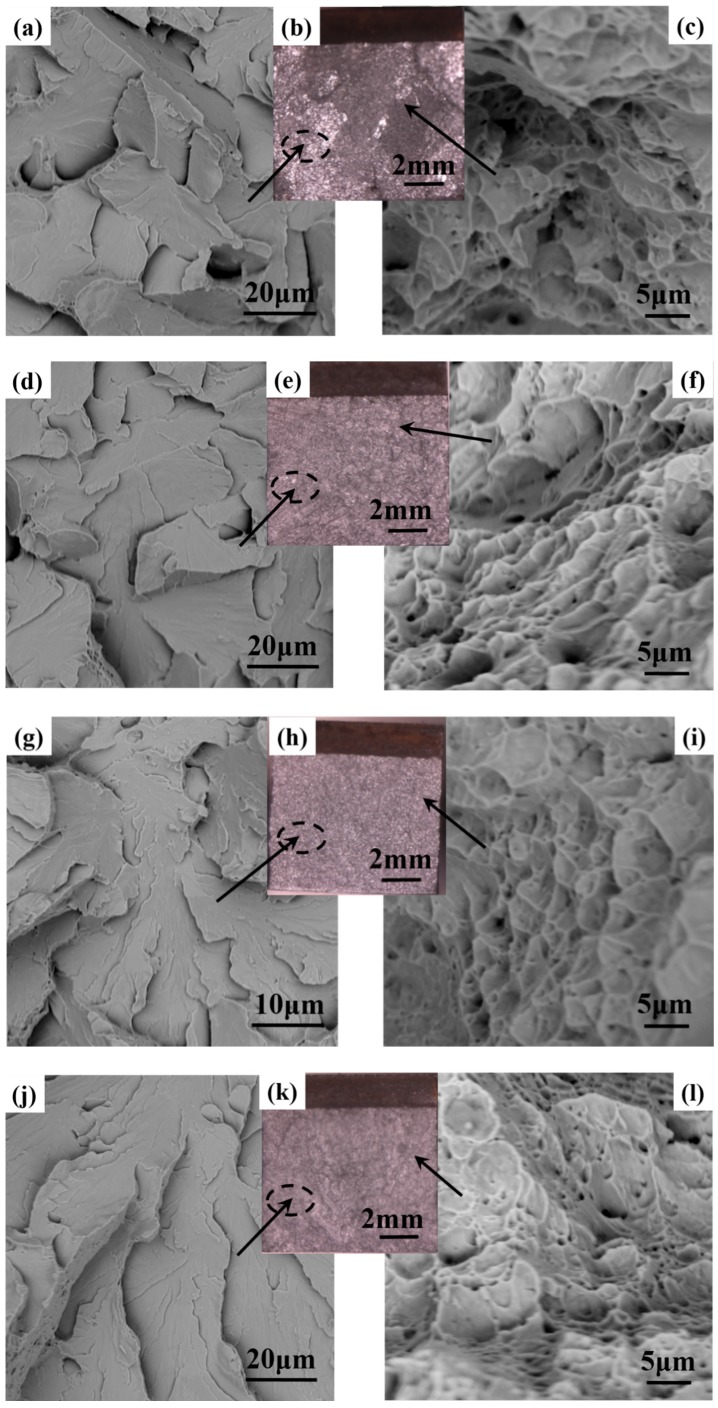
Fracture morphology features of specimens. (**a**–**c**) M1; (**d**–**f**) M2; (**g**–**i**) M3; and (**j**–**l**) M4.

Figure 12 shows the macro-fractographs and SEM images of the fracture surfaces of the four specimens away from the V-shaped notch. It can be clearly observed in Figure 12a–f that the fracture morphology was a mixture of quasi-cleavage fracture and ductile fracture, which was mainly composed of typical riverlike patterns and tongue patterns. In addition, dimples could also be found in the local region. The fibrous region and the secondary fibrous region can be clearly observed in Figure 12b,d. Figure 12g,i shows a ductile fracture characterized by microvoid coalescence, and the fracture surface can be divided into three regions: a fibrous region, a radial region, and a shear lip [37]. The size of unit cleavage facet became smaller and the number of dimples increased, contributing to the increase in crack propagation absorbed energy [10]. According to the formation mechanism of ductile fracture [38], the number of unit dimples reflects its ability to hinder the propagation of cracks. In addition, Figure 12i shows deeper dimples than Figure 12c,f, which implies that M3 had a higher toughness than M2 and M1. The fibrous region and riverlike patterns were apparently observed in M4, as shown in Figure 12j,l.

**Figure 13:**
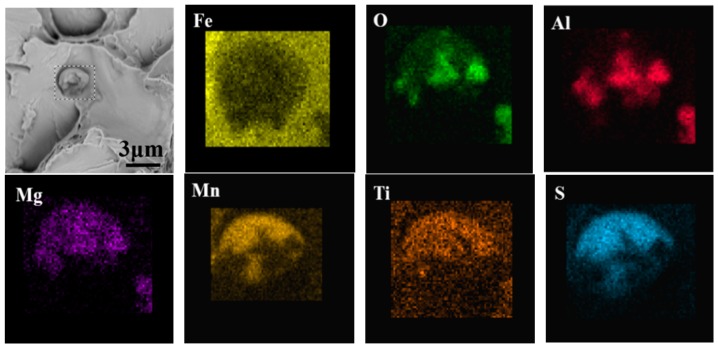
SEM images of the fibrous region on the fracture surface of the inclusion in M3.

Figure 13 shows SEM images of the fibrous region on the fracture surface of the inclusion in M3. The inclusions in the dimples were essentially spherical in shape and about 2 μm in size. The EDS analysis result indicates that the inclusions in M3 mainly consisted of MgO–Al_2_O_3_–TiO_2_–MnS. This type of appropriately sized inclusion can weaken the cleaving effect and reduce the stress concentration to improve toughness [39].

## 4. Discussion

### 4.1. Effect of Inclusions on the Mechanism of IAF at Different Isothermal Heat-Treatment Temperatures

**Figure 14:**
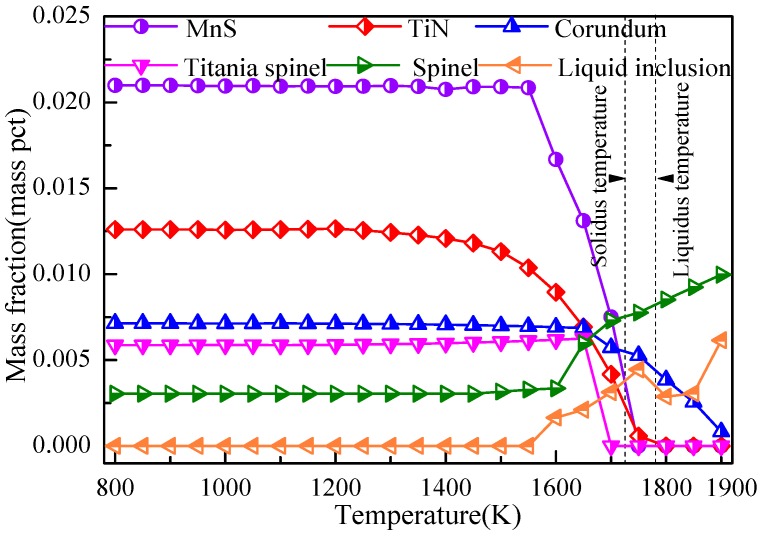
Phase transformation of inclusions in steel cooled from 1900 to 800 K.

To determine the capability of inclusions to induce IAF at different heat-treatment temperatures [40,41], it is necessary to predict the detailed precipitation behavior of inclusions to clarify the reason for the microstructural evolution. The mass fractions of the inclusion as a function of temperature were determined using FactSage7.1 software, as shown in Figure 14. According to the thermodynamic calculation results, the inclusions during solidification were corundum, spinel, titania spinel, TiN, and MnS. The corundum phase was a solid solution of TiO_2_ and Al_2_O_3_, while the spinel phase was composed predominantly of MgAl_2_O_4_. When the MgO nanoparticles mixed with AlTi nanoparticles were added to the experimental steel, MgO nanoparticles quickly dispersed within the melt, while Al and Ti dissolved in the liquid steel, which combined with dissolved oxygen to form corundum inclusions due to the strong deoxidizing element of Al and Ti. The strong stirring effect within the steel in the mold facilitated the formation of MgO–Al_2_O_3_–TiO_2_ inclusions. As the precipitation temperature decreased to be less than the solidus temperature of the experimental steel, MnS and TiN inclusions began forming on the surface of the oxide inclusions, and the Mn and Ti in the matrix-inclusion interfacial boundary layer were depleted, resulting in MDZ and transient zone formation near the inclusions [30,42]. Combining the above calculation results and Figure 6, the evolution behavior of these inclusions provides stable nucleation sites for IAF at heat-treatment temperatures from 973 to 823 K.

**Figure 15:**
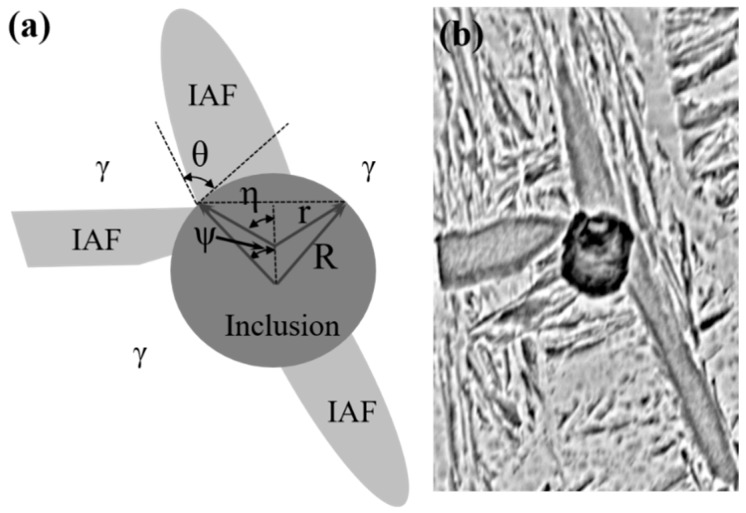
Model of IAF formation on the surface of a spherical inclusion. (**a**) Nucleation of IAF on the surface of inclusion, and (**b**) the actual SEM image of IAF nucleated on inclusion.

In addition to the composition of the inclusions discussed above, the size of the inclusions during the phase transformation was also an important factor affecting IAF. Therefore, the present work developed a modified method referring to previous models to predict IAF nucleation [43,44]. The calculation model for heterogeneous nucleation of IAF on the surface of inclusions was adopted as illustrated in Figure 15. Regarding the relation between the normalized energy barrier (${\Delta G}_{\mathrm{het}}^{*}$/${\Delta G}_{\mathrm{hom}}^{*}$) for IAF nucleation, the smaller the value of ${\Delta G}_{\mathrm{het}}^{*}$/${\Delta G}_{\mathrm{hom}}^{*}$, the easier IAF formation on the surface of inclusion [45]. Through a series of formula iterations, the inclusion size can be expressed as in Equations (1)–(4) [46,47]:

(1) $$\Delta G_{\mathrm{het}}^{*}/\Delta G_{\mathrm{hom}}^{*}=\frac{1}{2}+\frac{1}{2}\cos^{3}\eta +\frac{1}{2}\times {\left(\frac{r}{R}\right)}^{3}(2-3\cos\psi +\cos^{3}\psi )-\frac{3\cos\theta }{2}\times {\left(\frac{r}{R}\right)}^{2}(1-\cos\psi )$$

(2) $$\cos\eta =\frac{\frac{r}{R}-\cos\theta }{\sqrt{1-\cos^{2}\theta +{\left(\frac{r}{R}-\cos\theta \right)}^{2}}}$$

(3) $$\cos\psi =\frac{1-\frac{r}{R}\cos\theta }{\sqrt{1-\cos^{2}\theta +{\left(\frac{r}{R}-\cos\theta \right)}^{2}}}$$

(4) $$r_{K}=\frac{2\sigma_{\gamma \alpha }}{\Delta G_{V}}$$

where *r_K_* is the critical radius of IAF formation on the surface of inclusion (μm), *σ_γα_* is the interfacial energy between the austenite and ferrite phases (J/m^2^), ΔG*_V_* is the driving force for ferrite formation (J/m^3^), ${\Delta G}_{\mathrm{het}}^{*}$/${\Delta G}_{\mathrm{hom}}^{*}$ is the ratio of the energy barrier of homogeneous nucleation to the energy barrier of homogeneous nucleation, *ψ* and *η* are defined angle variables (deg), *r* is the radius of IAF (μm), *R* is the radius of the inclusion (μm), and *θ* is the contact angle between the ferrite and the surface of the inclusion (deg).

**Figure 16:**
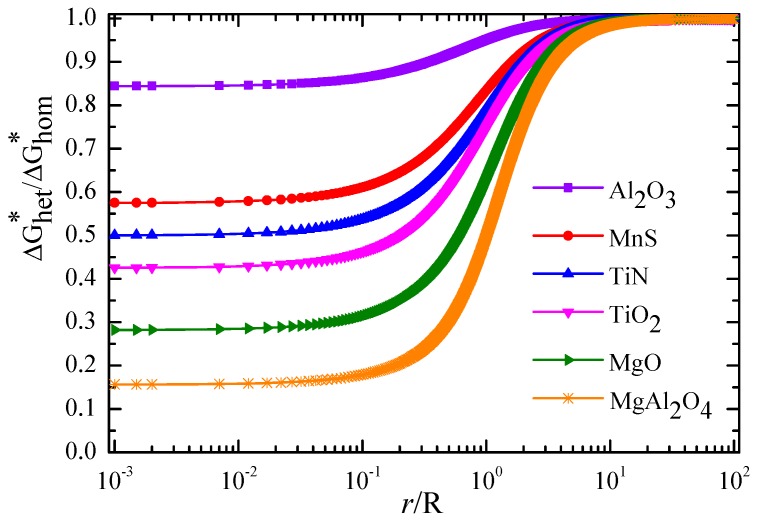
Effect of the value of *r*/*R* on energy barrier to ferrite at inclusions.

Combining Equations (1)–(3) with Table 3, the relationship between the value of *r*/*R* and the energy barrier to heterogeneous nucleation was calculated, as shown in Figure 16. It was found that ${\Delta G}_{\mathrm{het}}^{*}$/${\Delta G}_{\mathrm{hom}}^{*}$ of TiN, TiO_2_, MgO, and MgAl_2_O_4_ inclusions were less than 0.50, and the ${\Delta G}_{\mathrm{het}}^{*}$/${\Delta G}_{\mathrm{hom}}^{*}$ of MgAl_2_O_4_ inclusions was the smallest (about 0.16), while the values of the ${\Delta G}_{\mathrm{het}}^{*}$/${\Delta G}_{\mathrm{hom}}^{*}$ of MnS and Al_2_O_3_ inclusions were 0.57 and 0.85, respectively. Therefore, it can be inferred that the TiN, TiO_2_, MgO, and MgAl_2_O_4_ inclusions in the steel were effective for IAF nucleation, while the Al_2_O_3_ and individual MnS inclusions were not. In addition, the value of ${\Delta G}_{\mathrm{het}}^{*}$/${\Delta G}_{\mathrm{hom}}^{*}$ continuously decreased with decreasing the value of *r*/*R* and then tended toward a constant value. Hence, there was an optimal critical size for the inclusions inducing IAF nucleation. Judging from Figure 16, the critical value *r*/*R* ranged from 0.01 to 0.1.

**Figure 17:**
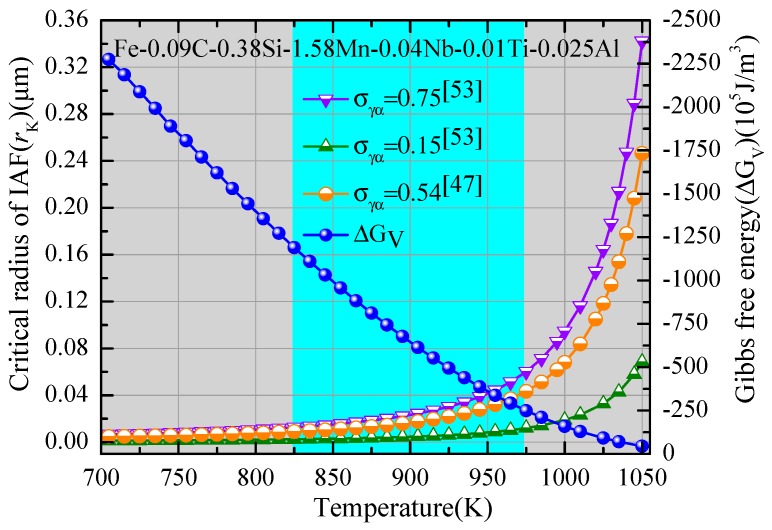
Relationship between the driving force and the temperature of the ferrite formation.

According to Equation (4) and ΔG*_V_* calculated in JMatPro software, it can be clearly seen (Figure 17) that the driving force (ΔG*_V_*) increased as the temperature decreased, which increased the capability for IAF nucleation on inclusions. Moreover, the critical radius of IAF decreased with the temperature decrease, and it was greatly affected by the value of *σ_γα_*. The critical radius of the IAF varied between 0.003 and 0.06 μm at a temperature ranging from 973 to 823 K. Therefore, the critical radius of inclusions ranged from 0.3 to 0.6 μm, and the values corresponding to critical diameters of inclusions were 0.6–1.2 μm. However, these calculation results were smaller than the actual inclusion size in the specimens, which may be due to these inclusions being large enough to become effective nucleation sites for IAF.

### 4.2. Refined Microstructure and Improved Mechanical Properties of Steel

**Figure 18:**
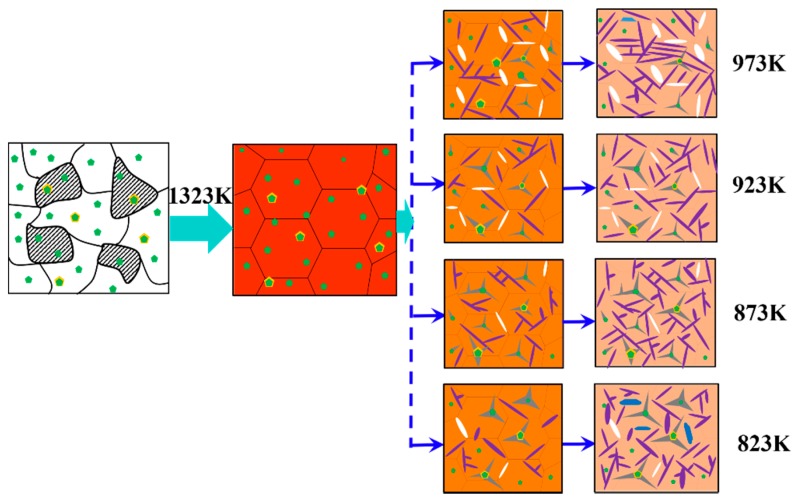
Schematic illustration of the refinement mechanism of the microstructure at different heat-treatment temperatures.

Combining the observations of the nucleation of IAF on inclusions in the four specimens, the refinement mechanism of the microstructure based on these findings is schematically illustrated in Figure 18. When the heat-treatment temperature was 973 K, it tended to form a large block of GBF and PF on the grain boundary because the prior austenite grain boundary had a high interfacial energy (Figure 9a). Byun et al. [45] reported that the inclusion surface was energetically less favorable than ferrite nucleation on the prior austenite grain boundaries at high temperatures. The current study results are similar to those, that is, a large amount of GBF nucleated on the grain boundary, resulting in a detrimental effect on toughness. As the temperature decreased, the microstructure varied from GBF and PF to IAF, and IAF began to nucleate at the boundary of the inclusion, while the IAF size at the beginning of nucleation was short. However, it is difficult to avoid GBF formation along the austenite grain boundary, which is the limiting factor for the further improvement of mechanical properties. When the temperature was decreased to 873 K, a larger number of IAF nucleated at the surface of complex oxide inclusions or between oxides and MnS, and then the SIAF grew on the IAF in different directions. Further, the inclusions promoted the formation of IAF plates within austenite grains and effectively inhibited GBF formation, which could divide the large austenite grains into many finer and separate regions (Figure 9c). The interlocking IAF formed during phase transformation could not only effectively refine the grains to improve the toughness of the steel but could also effectively prevent crack propagation [52], which is consistent with the results presented in Figure 12 in the current study. As the temperature dropped to 823 K, the inclusions still could induce IAF, but the IAF plates appeared to be significantly coarse (Figure 9d). At the same time, a small amount of bainite was also observed, indicating that the transformation of austenite into ferrite had been replaced by bainite. Based on the above discussion, it can be concluded that the optimal phase transformation temperature of IAF in specimens was about 873 K, which could refine the microstructure and relatively improve mechanical properties.

## 5. Conclusions

In this study, the characteristics and formation mechanisms of intragranular acicular ferrite in low-carbon steel were systematically investigated for different isothermal heat-treatment temperatures, and its influence on mechanical properties was also clarified. The following conclusions can be drawn.

1. After adding MgO nanoparticles predispersed with AlTi alloys to low-carbon steel, a large amount of finely dispersed MgO–Al_2_O_3_–TiO_2_ inclusions were formed in steel, and MnS or TiN inclusions precipitated on the surface of these oxide inclusions, which could act as nucleation sites of intragranular acicular ferrite.

2. By decreasing the heat-treatment temperature, the amount of grain boundary ferrite and polygonal ferrite was reduced, while the amount of intragranular acicular ferrite increased, and intragranular acicular ferrite that nucleated on the inclusions gradually grew up. However, intragranular acicular ferrite appears to be significantly coarsened and a small amount of bainite was also observed with the further temperature variations from 873 to 823 K.

3. The heat-treatment temperature had a slight effect on the strength of steel with MgO additions but had a significant effect on the average Charpy absorbed energy value, and the average Charpy absorbed energy value at 873 K was the maximum value, which showed excellent impact properties at room temperatures.

## Figures and Tables

**Table 1:** Chemical composition of experimental steel (wt. %).

C	Si	Mn	Nb	Ti	Ni	Al	S	N
0.09	0.38	1.58	0.04	0.01	0.25	0.025	0.008	0.003

**Table 2:** Mechanical properties of specimens.

Specimen	Yield Strength (MPa)	Tensile Strength (Mpa)	Elongation (%)
**M1**	535	769	9.6
**M2**	529	763	10.8
**M3**	531	803	13.6
**M4**	540	775	8.4

**Table 3:** The contact angle between the ferrite and the surface of the inclusion.

Inclusion	Contact Angle	cos*θ*	References
**Al_2_O_3_**	144	–0.809	[48]
**MnS**	118	–0.469	[22]
**TiN**	63	0.450	[22]
**TiO_2_**	52	0.616	[49]
**MgO**	35	0.819	[50]
**MgAl_2_O_4_**	31	0.857	[51]
